# To the Editors of the Medical and Physical Journal

**Published:** 1805-04-01

**Authors:** C. Simpson

**Affiliations:** Surgeon. Skipton


### *To the Editors of the Medical and Physical Journal.*

GENTLEMEN,

FROM your indulgence in inserting my case of compound fracture, (Medical and Physical Journal for October, 1803) I am induced to request you will allow me, through the medium of your widely extended and truly valuable publication, to return my *sincere thanks* to Mr. Cuming, for his recommendation of *nitre,* (Med. and Phys. Journal for April and Dec. 1804) in *cases of sphacelus.* I have recently had a case of that description, where every effort to relieve the patient proved fruitless, until I resolved upon the application of nitre; in which I was seconded by my very respectable and ingenious naval friend, Mr. Birtwhistle, of this place.

Having frequently experienced very good effects from the external application of crude sal. ammoniac finely powdered, in similar cases, and having previously formed a very high opinion of Mr. Cuming, from his style of writing, which I have repeatedly seen in your Journals, I was led to resolve upon giving a fair trial to nitre: I am happy in having it in my power to say the effect was favourable beyond my most sanguine expectation.

It would be superfluous to trouble you with the minutiæ of the case; suffice it to say, the whole class of stimulants and antiseptics were tried internally and externally; notwithstanding, the putrid diathesis increased daily, until arrested by this "sovereign remedy."

I shall conclude with joining Mr. Cuming, in strenuously recommending this invaluable medicine to the public; as one which, I flatter myself, will ultimately prove deserving their confidence and attention.

I am, &c.

C. SIMPSON, Surgeon.

*Skipton, Feb. 6,* 1805.

P. S. I shall, with your permission, at some future period, offer for insertion in your Journal a case of a very extensive and inveterate ulcer upon the leg, of thirty years standing.

